# The PI3K pathway regulates endochondral bone growth through control of hypertrophic chondrocyte differentiation

**DOI:** 10.1186/1471-213X-8-40

**Published:** 2008-04-11

**Authors:** Veronica Ulici, Katie D Hoenselaar, J Ryan Gillespie, Frank Beier

**Affiliations:** 1 CIHR Group in Skeletal Development and Remodeling, Department of Physiology & Pharmacology, University of Western Ontario, London, ON, N6A 5C1, Canada

## Abstract

**Background:**

The majority of our bones develop through the process of endochondral ossification that involves chondrocyte proliferation and hypertrophic differentiation in the cartilage growth plate. A large number of growth factors and hormones have been implicated in the regulation of growth plate biology, however, less is known about the intracellular signaling pathways involved. PI3K/Akt has been identified as a major regulator of cellular proliferation, differentiation and death in multiple cell types.

**Results and Discussion:**

Employing an organ culture system of embryonic mouse tibiae and LY294002, a pharmacological inhibitor of PI3K, we show that inhibition of the pathway results in significant growth reduction, demonstrating that PI3K is required for normal endochondral bone growth *in vitro*. PI3K inhibition reduces the length of the proliferating and particularly of the hypertrophic zone. Studies with organ cultures and primary chondrocytes in micromass culture show delayed hypertrophic differentiation of chondrocytes and increased apoptosis in the presence of LY294002. Surprisingly, PI3K inhibition had no strong effect on IGF1-induced bone growth, but partially blocked the anabolic effects of C-type natriuretic peptide.

**Conclusion:**

Our data demonstrate an essential role of PI3K signaling in chondrocyte differentiation and as a consequence of this, in the endochondral bone growth process.

## Background

Bone formation occurs through two different mechanisms: endochondral and intramembranous ossification. Longitudinal growth of the axial and appendicular skeleton is a result of endochondral ossification (EO) that is controlled by the cartilage growth plate [1]. EO involves the aggregation of mesenchymal cells to form cartilaginous nodules [2]. A subset of the cells in these nodules matures further into growth plate chondrocytes.

During endochondral bone development in the limb, growth plate chondrocytes undergo well-ordered and controlled phases of cell proliferation, maturation, and apoptosis [3]. The growth plate can be divided into three main chondrocyte subpopulations: the resting, proliferating and hypertrophic chondrocytes. These populations are arranged in distinct zones that are distinguishable by morphological criteria, but are also characterized by specific molecular markers. The proliferation and/or differentiation of these subpopulations are controlled by a complex network of regulatory molecules [4]. Proliferative chondrocytes synthesize type II collagen and form characteristic columns; they then exit the cell cycle and become post-mitotic prehypertrophic chondrocytes that differentiate further into hypertrophic cells. Hypertrophic chondrocytes express type X collagen and mineralize the surrounding matrix. This differentiation process is followed by apoptosis of hypertrophic chondrocytes, but prior to their death, they deposit vascular endothelial growth factor (VEGF) into their extracellular matrix, which promotes the invasion of blood vessels into the cartilage tissue. Blood vessel invasion enables the recruitment of osteoblasts and osteoclasts and replacement of the cartilage scaffold by a calcified bone matrix [2-5]. This final step results in the formation of trabecular bone (the primary spongiosa). With continuing resorption of the primary spongiosa by osteoclasts, the primary center splits into two opposing growth plates, in each of which the maturation of cartilage and subsequent remodeling into bone continue, as long as new chondrocytes are generated in the growth plates [6].

Hypertrophic chondrocytes play a pivotal role in coordinating chondrogenesis and osteogenesis, as they provide a scaffold for subsequent formation of trabecular bone and secrete factors such as VEGF that control the activity of other cells involved in EO. Therefore, the proper regulation of chondrocyte differentiation and the coordination of chondrocyte progression through the cell cycle have to be tightly regulated for normal bone growth. The induction of growth arrest is a central feature of this phenotypic transition. For example, mice lacking the cyclin dependent-kinase inhibitor p57/Kip2 exhibit several developmental abnormalities including abnormal skeletogenesis [7]. Moreover, numerous skeletal diseases are caused by deregulation of cellular proliferation and hypertrophic chondrocyte differentiation, such as a large number of skeletal dysplasias that are characterized by dwarfism, skeletal deformities, and frequently by early-onset osteoarthritis [8].

Both local paracrine regulators and systemic hormones control endochondral bone formation and bone remodeling throughout life. Insulin-like growth factor-I (IGF1) and C-type natriuretic peptide (CNP) are among the major stimulators of endochondral bone growth. IGF1 is the most prominent growth factor involved in linear growth regulation and it was shown to be essential for growth plate chondrocyte development. The most prominent effect of IGF1 is induction of chondrocyte hypertrophy, as shown both in IGF1 null mice and in bone cultures treated with IGF1 [9-12]. In addition, studies from our lab and others identified the CNP pathway as an important anabolic regulator of endochondral bone growth [13-15]. However, the molecular and cellular mechanisms mediating the anabolic effects of both ligands are not completely understood. Substantial progress has been made in the past few years in understanding how local signaling molecules, working through key transcription factors such as Sox and Runx proteins, interact and control the growth and differentiation of bones [3,5,16-18]. However, the intracellular signaling pathways connecting extracellular signaling molecules to transcriptional regulators are poorly understood.

Here we focus on Phosphatidylinositol 3-kinases (PI3Ks) which represent a family of lipid kinases whose inositol lipid products are key mediators of intracellular signaling in many cell types [19]. PI3Ks are represented by a family of eight distinct enzymes that can be divided into three classes based on their structure and function [20]. Class I PI3Ks have been the major focus of PI3K studies because these isoforms are generally coupled to extracellular stimuli. The generation of D3-phosphorylated phosphoinositides at the membrane by PI3Ks results in the recruitment of certain signaling proteins to the plasma membrane via their pleckstrin homology (PH) domains. As such, PI3Ks are upstream regulators in a number of signaling cascades that control proliferation, growth, cell death, migration, metabolism, and a host of other biological responses [20]. In addition, the PI3K pathway is known as the major signaling cascade downstream IGF1 in many cell types [21-24].

Class I PI3Ks are reversibly inhibited by the pharmacological compound LY294002, and more specifically class I alpha isoforms by PI3-K α inhibitor IV from Calbiochem. Genetic screens in model organisms have identified Akt (protein kinase B) as the primary downstream mediator of the effects of PI3K [25]. PtdIns (4, 5)*P2 *and PtdIns (3, 4, 5)*P3 *bind to the PH domain of Akt, recruiting the kinase to the plasma membrane where Akt is phosphorylated and activated [26-28]. Akt has been shown to be a critical mediator of cell proliferation and survival [29]. In mice, disruption of the most ubiquitously expressed member of the *Akt *family of genes, *Akt1*, results in body size reduction compared to the wild-type littermates [30,31].

Therefore, the PI3K/Akt pathway is involved in proliferation, differentiation, cellular survival or a combination of these processes in multiple cell types, from neurons to fibroblasts [32] but its role in cartilage and bone development has not been studied intensively and is the focus of our study.

## Results

### LY294002 suppresses chondrocyte differentiation

**Figure 1:**
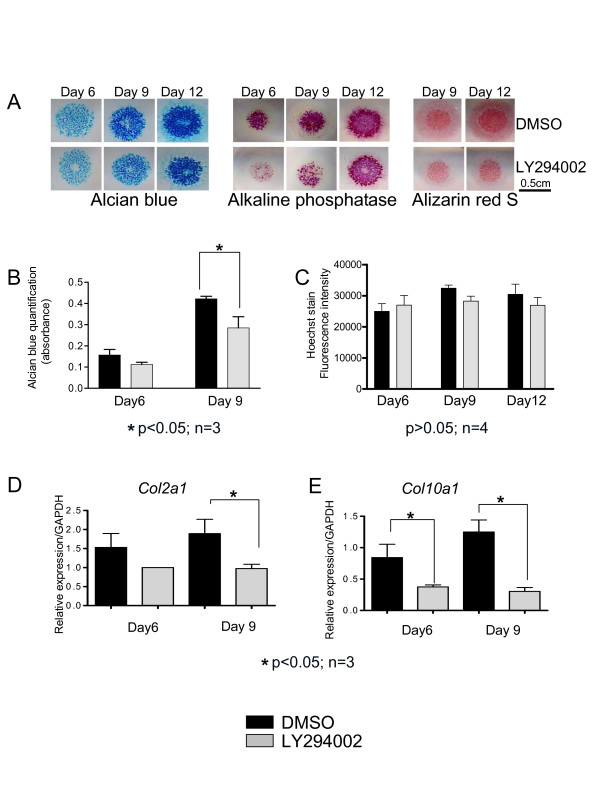
**PI3K inhibition results in reduced chondrocyte differentiation**. (A) Mesenchymal cells isolated from E11.5 mouse limb buds were cultured for 3 days and then treated with LY294002 or DMSO as vehicle control. They were stained after 6, 9 and 12 days, respectively for different chondrocyte differentiation markers: Alcian blue for glycosaminoglycans, Alkaline phosphatase activity and Alizarin red for the calcium content. The intensity of these markers is reduced in the LY294002 treated cultures. (B) After 9 and 12 days respectively the Alcian blue content of the micromasses was spectrophotometrically measured at 620 nm, after extraction with 6 M Guanidine hydrochloride. We noticed decreased absorbance levels for the LY294002 treated cultures. (C) Measurements of Hoechst fluorescence intensity (excitation/emission: 350/450 nm) showed no significant difference in the DNA content between the LY294002 and DMSO treated micromass cultures. (D, E) RNA was isolated from the micromass cultures after 6 and 9 days of culture and real time analysis was performed. The relative transcript levels for *col2a1 *and *col10a1 *were decreased in the LY294002 treated micromasses compared to the vehicle control.

Micromass cultures were incubated in medium for three days to allow chondrogenic differentiation before addition of the PI3K inhibitor LY294002 (10 μM) or DMSO (Dimethyl Sulfoxide, control) for up to 9 more days (e.g. until 12 days of culture). PI3K inhibition caused a delay in chondrocyte differentiation, as shown by decreased accumulation of sulfated glycosaminoglycans (Alcian blue stain), decreased mineralization (Alizarin red S) and less staining for alkaline phosphatase activity compared to cells treated with DMSO (Figure 1A). Alcian blue stain was extracted and quantified spectrophotometrically, confirming a significant decrease in LY294002 treated micromass cultures at day 9 (Figure 1B). Measurement of Hoechst 33342 fluorescence demonstrated that LY294002 treatment did not result in significant changes in DNA content of cultures (Figure 1C). RNA was isolated from the micromass cultures at days 6 and 9 of culture. Real-time PCR experiments showed decreased relative levels of collagen II (Figure 1D) and X (Figure 1E) transcripts upon PI3K inhibition. These data demonstrate that PI3K activity is required for normal progression of chondrocyte differentiation.

### PI3K inhibition results in reduced bone growth

**Figure 2:**
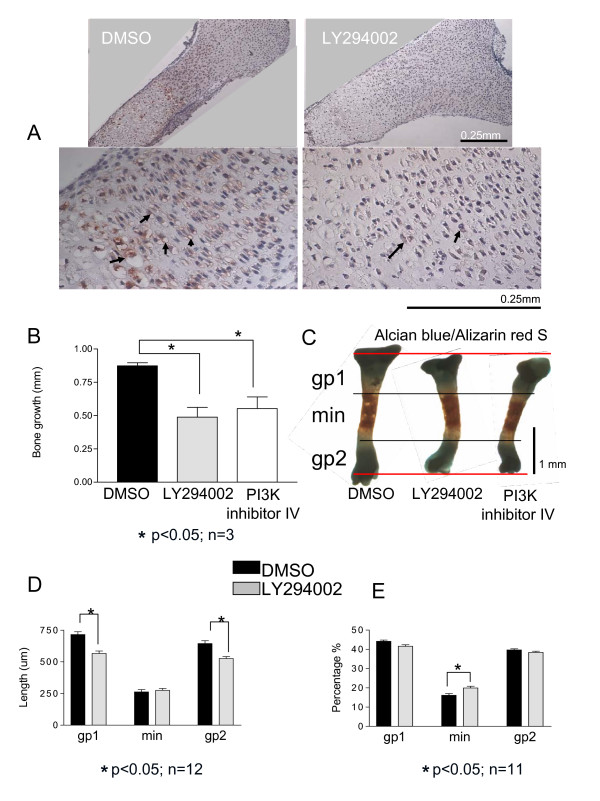
**Decreased growth of the LY294002 treated tibiae**. (A) E 15.5 mouse tibiae, cultured for 6 days in the presence of LY294002 or DMSO, were analyzed by immunohistochemistry, at the end of the time course. Phosphorylation of Akt at Ser473 was found to be reduced under PI3K inhibition with LY294002 (10 μM). Black arrows show cells expressing phosphorylated Akt. (B) The difference between the length of the tibiae in the beginning and at the end of the time course represents the bone growth and it was found to be significantly reduced in bones treated with LY294002 (45% reduction) or PI3-K α inhibitor IV (500 nM) (35% reduction) compared to DMSO. (C) At the end of culture period the tibiae were also stained with Alcian blue/Alizarin red. We notice decreased length of both proximal (gp1) and distal (gp2) growth plates of tibiae treated with LY294002 or PI3-K α inhibitor IV. (D) Measurements of the growth plates and mineralized area (min) length confirmed the reduction of these under PI3K inhibition with LY294002. (E) When the gp1, gp2 and min were expressed as ratio of the entire bone length there is an increased ratio of the mineralized area in the LY294002 treated tibiae.

We next investigated the role of PI3K/Akt signaling in the three-dimensional context of an intact bone. Tibiae were isolated from E 15.5 mice, measured and then incubated with LY294002 (10 μM), PI3-K α inhibitor IV (500 nM) or DMSO for six days. Immunohistochemistry demonstrated that in control cultures, phosphorylated Akt (P-Akt) proteins (indicating activity of the pathway) can be found mostly in the late proliferative, prehypertrophic and early hypertrophic areas (Figure 2A). P-Akt levels were greatly reduced upon PI3K inhibition, demonstrating the efficiency of the inhibitor. The difference between tibia length in the beginning and at the end of the time course represents bone growth. The bones treated with LY294002 and PI3-K α inhibitor IV showed an average of 45% and 35% reduction in growth compared to tibiae treated with DMSO (Figure 2B). Measurements of both growth plates and the mineralized area showed that the length of the two tibial growth plates (proximal and distal) was reduced in the LY294002 treated bones, whereas the absolute length of the mineralized zone was not affected (Figure 2C and Figure 2D). PI3-K α inhibitor IV showed similar effects to LY294002 as observed in the Alcian blue/Alizarin red stain (Figure 2C).

When the three parts of the bone (gp1-proximal growth plate, gp2-distal growth plate, and min-mineralized area) were calculated as a percentage of the entire length of the bone, we noticed a relative increase in the length of the mineralized zone upon PI3K inhibition (Figure 2E).

### Treatment of tibiae with LY294002 results in smaller proliferative and hypertrophic zones

**Figure 3:**
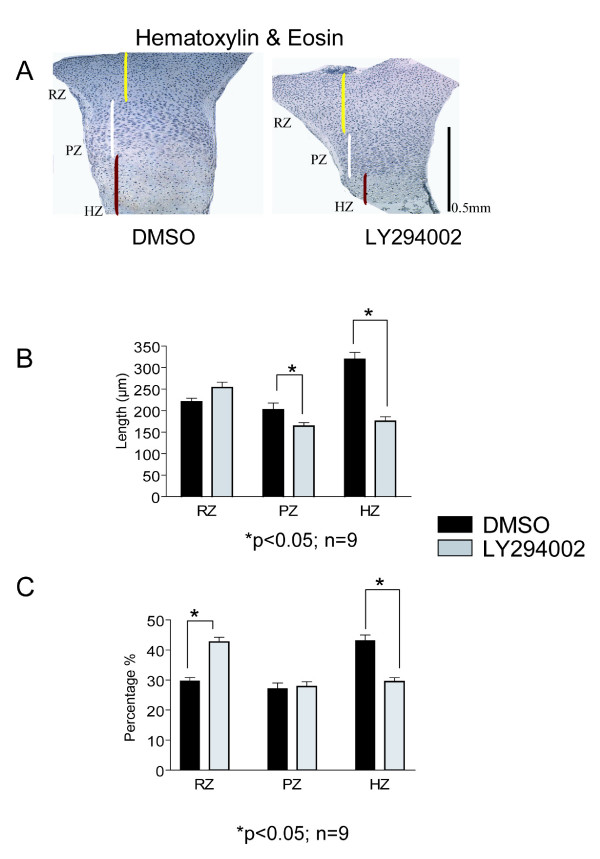
**Reduced length of the proliferative and hypertrophic zones under PI3K inhibition**. (A) E15.5 tibiae treated for 6 days with LY294002 or DMSO were fixed, paraffin embedded and sectioned. Hematoxylin & eosin (H&E) staining shows decreased length of hypertrophic (HZ) and proliferative zones (PZ) and increased resting zone (RZ) length in the LY294002 treated bones. (B) Measurements of three zones of the growth plate confirmed the reduction in length for the HZ and PZ in the LY294002 treated tibiae, but the RZ length zone was not found to be significantly increased. (C) The HZ length expressed as ratio from the entire growth plate length was found to be significantly decreases and the RZ significantly increased in the case of LY294002 treatment. The PZ ratio was found to be similar between the treatments.

Histological sections of tibiae from E15.5 mice, cultured for 6 days in the presence of LY294002 (10 μM) or DMSO, were stained with H&E or Safranin O/Fast green. Growth plate organization and columnar arrangement of cells were not notably different after PI3K inhibition, but we noticed a much smaller hypertrophic zone in LY294002 treated bones compared to the controls (Figure 3A). Sections were further analyzed using Openlab 4.0.4 software to measure the length of different areas of the growth plate. The hypertrophic (HZ) and proliferative (PZ) zones of the growth plate of LY294002 treated tibiae were significantly shorter than in control. The resting zone (RZ) was increased in length in LY294002 treated bones, but this was not found statistically significant (Figure 3B).

Similar results were found when we calculated the length of the zones reporting the measurements as percentage of the entire growth plate, but in this case the decrease in proliferative zone length was not found to be statistically significant (Figure 3C). Since LY294002 had similar effects on the proximal or distal growth plates (Figure 2C and Figure 2D), zone measurements were performed on the proximal growth plate only.

### Treatment of tibiae with LY294002 results in reduced hypertrophic cell size

**Figure 4:**
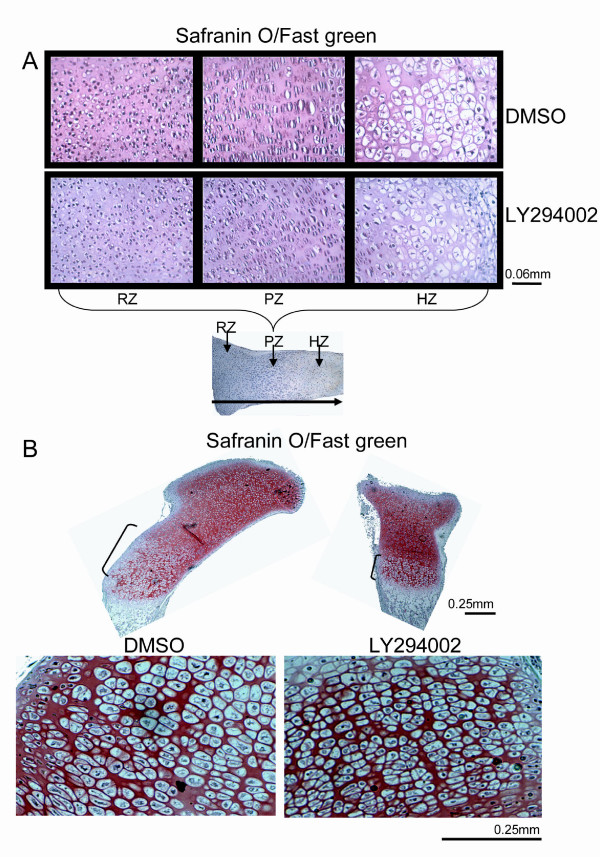
**PI3K inhibition affects chondrocyte morphology**. (A) E15.5 tibiae, cultured for 6 days in the presence of LY294002 or DMSO were stained with Safranin O for proteoglycan content. The chondrocyte morphology was analyzed by comparing the chondrocyte size in the three zones of the growth plate. We noticed a reduction of hypertrophic cell size in the case of hypertrophic chondrocytes. (B) E18.5 tibiae treated with LY294002 for 6 days showed the same reduction in hypertrophic zone length and cell size as the E15.5 treated tibiae.

We next analyzed the effects of LY294002 on chondrocyte morphology. While resting and proliferative cells did not display obvious differences in cell size or shape under the different conditions, cells in the hypertrophic zone appeared markedly smaller upon PI3K inhibition (Figure 4A).

To examine whether the observed effects were specific for the chosen developmental stage, we performed similar experiments with tibiae from E18.5 mice. Tibiae cultured for 6 days in the presence of LY294002 demonstrated decreased length of the growth plate and the hypertrophic zone, similar to E15.5 tibiae. We also noticed decreased size of hypertrophic cells in the presence of the PI3K inhibitor (Figure 4B), demonstrating that the anabolic role of the PI3K pathway is not specific to a certain developmental stage.

### Decreased markers of chondrocyte differentiation and increased apoptosis in LY294002 treated growth plates

**Figure 5:**
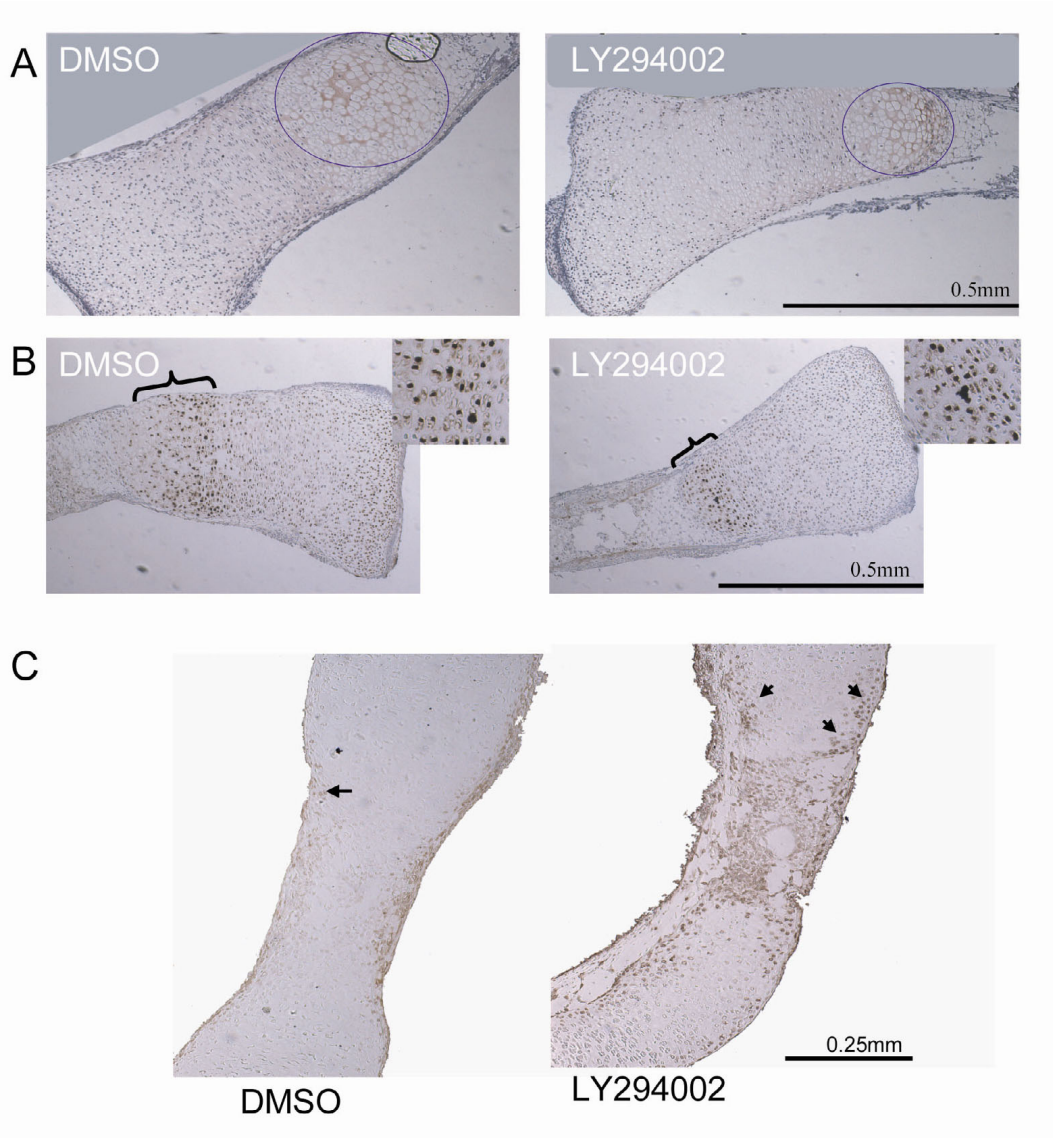
**Decreased markers of chondrocyte differentiation and increased apoptosis in the LY294002 treated tibiae**. (A) E15.5 tibia organ cultures were analyzed by immunohistochemistry for collagen X expression. The hypertrophic cell area (marked by dashed blue circles), which showed positive collagen X stain, was smaller in the LY294002 treated bones. (B) Tibiae were also analyzed by immunohistochemistry for cyclin-dependent kinase inhibitor 1C (p57), a marker of cell cycle arrest in G1 and chondrocyte differentiation. There was a narrower area of p57 positive cells (showed by brackets) in the LY294002 treated bones. (C) E 15.5 tibiae treated for 6 days with LY294002 shoed an increased number of apoptotic cells in the hypertrophic and mineralized areas (black arrows) as shown by colorimetric TUNEL assay.

We next examined molecular markers of chondrocyte differentiation in E15.5 tibiae cultured for 6 days in the presence of LY294002 or DMSO, using immunohistochemistry. The domain of collagen X staining was decreased upon PI3K inhibition (Figure 5A), in agreement with the smaller hypertrophic zone observed under these conditions. In addition, expression of cyclin-dependent kinase inhibitor p57 (Kip2), a marker of postmitotic chondrocytes, was decreased in the LY294002 treated bones, providing further evidence for decreased and delayed chondrocyte differentiation (Figure 5B).

We next examined the effects of PI3K inhibition on chondrocyte proliferation and apoptosis. BrdU (Bromodeoxyuridine) labeling revealed no significant difference in the percentage of replicating cells within the proliferative zone of the growth plate (data not shown). Treatment of E15.5 tibiae with LY294002 or DMSO for 6 days results in increased number of cells showing positive TUNEL stain in the hypertrophic zone of LY294002 treated bones (Figure 5C).

### IGF1-induced bone growth partially requires PI3K activity

**Figure 6:**
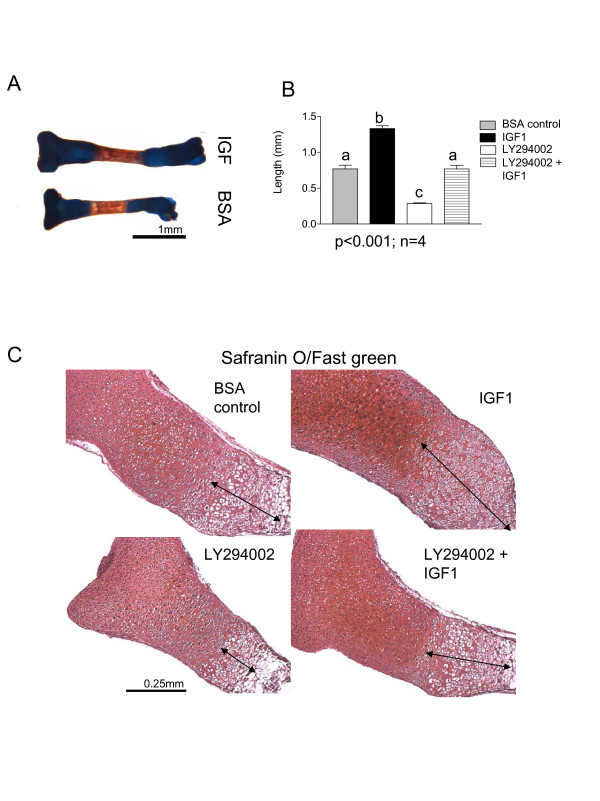
**IGF1-induced bone growth partially requires PI3K activity**. (A)Tibiae isolated from E15.5 mice were cultured for 6 days in the presence of IGF1 or 0.1% BSA in PBS, as control. IGF1 induced significant increase in bone growth. (B) Measurements of bone growth after 6 days of treatment with IGF1, control, LY294002 or IGF1 + LY294002 showed increased bone growth in the IGF1 treatment, and similar bone growth between the control and the IGF1 + LY294002 treatment. (C) E 15.5 tibiae treated for 6 days with the treatments mentioned above, were fixed, embedded, sectioned and stained with Safranin O. The length of hypertrophic zone was increased in the IGF1 treatment compared to control; decreased in the LY294002 treatment as shown before and in the case of the IGF1 + LY294002 treatment similar to the control hypertrophic zone length.

We next attempted to identify extracellular signals that control bone growth through the PI3K pathway. IGF1 is a known anabolic factor for endochondral bones [12] and has been shown to activate PI3K signaling [22,24]. Tibiae isolated from E15.5 mice were cultured in the presence of IGF1 (50 ng/ml of medium), LY294002, control (0.1% Bovine serum albumine (BSA) in PBS) or IGF1+LY294002. IGF1 treatment caused a significant increase in bone growth (around 70%) (Figure 6A). As before, LY294002 treatment resulted in more than 45% reduction in growth. However, IGF1 stimulated bone growth to a similar degree in LY294002 treated bones (Figure 6B), with the ratio between the bone growth in the IGF1 treatment and control (1.73) being lower that the ratio between the LY294002+IGF1 treatment and LY294002 (2.67). There is no significant difference between the control and LY294002+IGF1 treatment. This suggests that in addition to PI3K, another pathway is required for IGF1-induced bone growth. Histological investigation demonstrated that IGF1 induced enlargement of the hypertrophic zone in the absence and presence of LY294002, as shown by increased hypertrophic zone length in IGF1 treatment compared to control and in LY294002+IGF1 treatment compared to LY294002 alone (Figure 6C).

### C-type natriuretic peptide-induced bone growth requires PI3K activity

**Figure 7:**
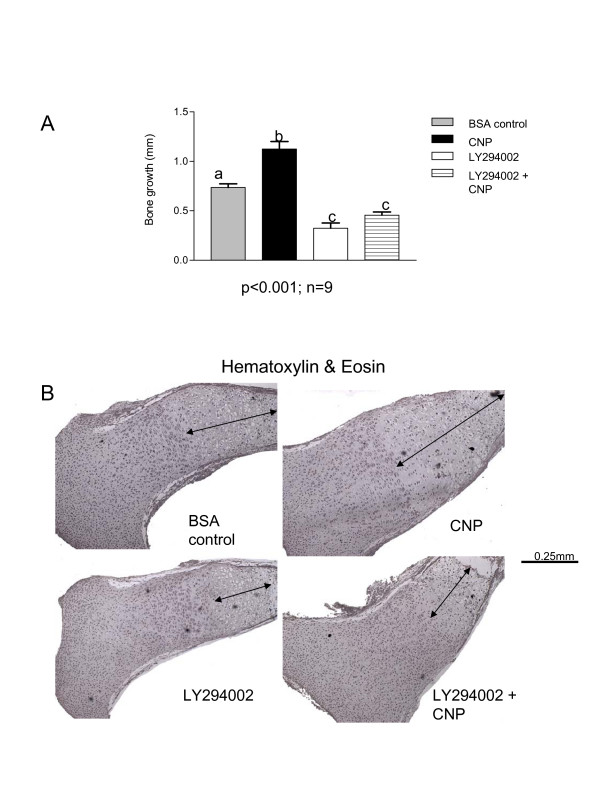
**CNP-induced bone growth requires PI3K activity**. (A) Tibiae isolated from E15.5 mice were cultured for 6 days in the presence of CNP, control (0.1% HCl-BSA in PBS), LY294002 or CNP + LY294002. Bone growth measurements showed increased bone growth in the CNP treatment, and no significant difference between the LY294002 and CNP+LY294002. (B) E 15.5 tibiae treated for 6 days with the treatments mentioned above, were fixed, embedded, sectioned and stained with H&E. The length of hypertrophic zone was increased in the CNP treatment compared to control, decreased in the LY294002 treatment and similar between LY294002 and CNP+LY294002

We next turned our attention to CNP, another potent stimulator of bone growth [33]. Tibiae were isolated from E15.5 mice and treated with control (0.1% HCl-BSA in PBS), CNP (10^-6 ^M), LY294002 or CNP+LY294002. CNP strongly stimulated bone growth in the absence of LY294002. Upon PI3K inhibition, CNP-induced bone growth is blocked (Figure 7A), with no significant difference between the LY294002 and LY294002+CNP treatments. Similarly to IGF1, CNP induces an enlargement of the hypertrophic zone but in this situation it seems to be dependent on PI3K activity, as shown by reduction of the hypertrophic zone length in the CNP+LY294002 treatment almost to the level of the LY294002 treatment (Figure 7B).

## Discussion

The PI3K pathway has been shown to affect numerous cellular processes in a tissue-specific fashion; for example, it is required for survival in different cell types such as cardiomyocytes [34], cellular differentiation in the case of osteoclasts and keratinocytes [35,36], and proliferation and differentiation of osteoblasts [35]. It also stimulates differentiation of CD4+ T-cells [37] and development and proliferation of B cells [38,39]. We hypothesized that the PI3K pathway has similar effects in the growth plate, promoting endochondral bone growth by increasing proliferation and differentiation of chondrocytes and by suppressing apoptosis.

We found that inhibition of PI3K with LY294002 results in decreased differentiation, in both primary chondrocytes (micromass cultures) and organ cultures. Markers of both early chondrocyte differentiation such as collagen II and glycosaminoglycans and of late hypertrophic differentiation such as collagen X, p57, Alkaline phosphatase activity and calcium content (Alizarin red S stain) were decreased upon PI3K inhibition. These data suggest that the PI3K pathway is required for normal chondrocyte differentiation. In the organ culture system, we have shown that the PI3K pathway is required for maximal bone growth, since by inhibiting the pathway we obtained 55% reduction in bone growth, due to a proportionate shortening of both growth plates.

The major phenotype of the LY294002 treated tibiae is represented by a 45% reduction in the length of the hypertrophic zone, providing further evidence that the PI3K pathway is required for hypertrophic differentiation. The observed reduction in the area staining for collagen X and p57 in LY294002 treated tibiae is in agreement with reduced hypertrophy. In addition we have seen a 20% reduction of the length of the proliferative area of the growth plate, in LY294002 treated tibiae. In the organ culture system it seems that the onset of proliferation is delayed, since the resting zone represents a higher percentage of the growth plate in the LY294002 treated bones compared to control. The ratio of BrdU labeled cells within the proliferative zone of the growth plate does not appear to be different between LY294002 and control cultures, suggesting that PI3K inhibition results in delayed cell cycle entry, but does not affect the rate of cell cycle progression once cells have entered to proliferative zone. Our data also show increased apoptosis in organ cultures treated with LY294002. Apoptosis was only detected in the hypertrophic and mineralized zones, suggesting that the PI3K pathway is required for hypertrophic chondrocyte survival.

PI3K signaling transduces signals from many growth factors and other extracellular cues, but it is not known which of them utilizes the pathway for anabolic effects on endochondral bones. Potential candidates are IGFs, however, our data suggest, somewhat unexpectedly that IGF1 stimulates organ culture growth in the presence of LY294002 to a similar degree as in control cultures. IGF1 treatment causes an increase in the length of hypertrophic zone [11], and this increase is not completely blocked by the PI3K inhibitor. This suggests that the PI3K pathway is not the only and potentially not the major pathway required for IGF1-induced bone growth and -hypertrophic differentiation in our organ culture system.

One potential problem that could partially explain the lack of growth reduction in the IGF1 + LY294002 treatment is that IGF1 possibly increased Akt phosphorylation to a level that is no longer completely inhibited by 10 μM LY294002. The mechanisms for IGF1 and CNP regulation of the PI3K pathway in growth plate chondrocytes are not the focus of this manuscript, but we plan to investigate the implications of these two growth factors in more depth in future studies. It will be important to see the levels of phosphorylated Akt in all treatment combinations, by performing immunohistochemistry and Western blotting with protein isolated directly from the tibiae treated with all treatment combinations (control, IGF1, LY294002 and LY294002 + IGF1 or control, CNP, LY294002, and CNP +LY294002). In addition, future measurements of growth plate zones under all conditions might provide an explanation for the sustained anabolic effects of IGF1even in the presence of LY294002. Our results have shown that the PI3K pathway is mostly involved in hypertrophic chondrocyte differentiation, and also that the two ligands IGF1 and CNP increase the length of hypertrophic zone (Figures 6C and 7B). There were no obvious effects on the other zones, but performing growth plate zone measurements and molecular analyses (e.g. BrdU labeling) might bring additional information in the future.

It will also be of interest to determine which other pathways mediate anabolic activities of IGFs in cartilage; the other major signaling pathway implicated in IGF signaling in other cells, the MEK-ERK (mitogen-activated protein kinase kinase/extracellular regulated kinase) cascade, has been shown to suppress endochondral bone growth [33,40-42] and is therefore an unlikely candidate for this role.

Surprisingly, C-type natriuretic peptide (CNP), which is not a known PI3K activator, was found to partially require PI3K activity to stimulate bone growth. The CNP-induced growth of cultured tibiae was blocked by the PI3K inhibitor. One interesting finding was that the effect of CNP on hypertrophy – a significant increase in the hypertrophic zone length [13] – was inhibited by LY294002. These data identify CNP as one signal requiring PI3K activity in cartilage, but there are other potential candidates for regulation of the PI3K pathway in endochondral bone growth, such as PTHrP (Parathyroid hormone-related protein) and integrin ligands. Studies are under way in our laboratory to identify physiological activators of PI3K signaling in cartilage.

The molecular mechanisms mediating the effects of PI3K signaling in endochondral bone growth remain to be identified. We show that Akt proteins are phosphorylated under control conditions, and that this activation is reduced under PI3K inhibition, resulting in reduced bone growth, in agreement with reduced growth in *Akt1*-deficient mice as well as mice deficient in multiple *Akt *genes[43,44]. The PI3K/Akt pathway was shown to be involved in Runx2 (runt related transcription factor 2) -dependent osteoblast and chondrocyte differentiation in 2 cell lines, MC3T3-E1 and ATCDC5, respectively [45]. Therefore it represents a candidate for the PI3K involvement in chondrocyte hypertrophy. Further investigations of the PI3K/Akt mechanisms of chondrocyte differentiation are necessary in order to find the direct targets of this signaling pathway.

## Conclusion

We have shown that PI3K is required for normal growth plate chondrocyte differentiation and survival in vitro, and therefore for endochondral bone growth. Future studies are required to further analyze the mechanisms by which PI3K exerts these effects, investigating both the molecules downstream of PI3K and the upstream activators of the pathway, and the mechanisms used by these molecules in order to function within the PI3K/Akt pathway.

## Methods

### Materials

Timed pregnant CD1 mice were purchased from Charles River Laboratories. Cell culture and organ culture medium components and general chemicals were purchased from Sigma and Invitrogen. LY294002, PI3-K α inhibitor IV, and the TdT-FragEL™ DNA Fragmentation Detection Kit were purchased from Calbiochem, Antibodies for immunohistochemistry were purchased from Sigma (monoclonal anti-collagen type X-C7974), Cell Signaling Technology^® ^(P-Akt (Ser 473) #4051; Akt #9272), Santa Cruz Biotechnology^® ^(Kip2 p57 (H-91)). Hoechst 33342 nuclear acid stain was purchased from MolecularProbes; Pronase E, used for antigen retrieval in immunohistochemistry, from Sigma (#P5147) and AEC substrate-chromogen was purchased from Dako.

### Micromass culture

Mouse embryos were dissected at E11.5 and mesenchymal cells were isolated from limb buds by digestion for 90 minutes in dispase as described [46]. Cells were resuspended in medium containing 60% F12, 10% FBS, 0.25% L-glutamine and 0.25% Penicillin-Streptomycin and plated at high density (2.5 × 10^7 ^cells/ml) in 10 μl droplets, to stimulate high density cellular contacts. The cells were cultured for up to 12 days, and the medium was supplemented with 2 μl beta-glycerophosphate (final concentration 1 mM) and 20 μl ascorbic acid (final concentration 0.25 mM) for each ml of medium. Medium was changed daily [46]. Cells were differentiated in micromass culture for 3 days to allow chondrogenesis to occur before addition of LY294002 (10 μM) or DMSO and staining with Alcian blue or Alizarin red S and for alkaline phosphatase activity, as described [46-48]. Alcian blue-stained micromass cultures were incubated with 500 μl of 6 M Guanidine hydrochloride over night to extract the stain, as described [48]. The absorbance of the Alcian blue solution was measured at 620 nm.

### Measurement of DNA content in micromass cultures using Hoechst staining

The UV-excitable DNA stain Hoechst 33342 at final concentration of 5 μg/ml was used to quantify DNA content in the micromass cultures. Micromass cultures from the same trials used in the Alcian blue, Alizarin red and alkaline phosphatase stains, plated in parallel wells, were used for this experiment. Culture times, treatments and fixation procedures were all carried out in the same conditions as for the above mentioned stains. Cells were then incubated with Hoechst DNA dye for 15 minutes, washed with PBS and trypsinized 2 × 10 minutes at 37°C. The cells were then centrifuged at 1000 rpm for 2 minutes and re-suspended in culture medium. Re-suspended cells were used to measure the DNA content in these cultures using a fluorimeter (Model RF-M2004, Photon Technology International, London, ON) with excitation at 350 nm and emission at 450 nm. Data from 3 different trials was analyzed using Felix32 Software.

### RNA isolation and real-time PCR

RNA was isolated from micromass cultures as previously described [49]. Taqman real-time PCR was performed to quantitatively asses RNA samples [46,48,49] with primers and probe sets from Applied Biosystems; data were normalized to *Gapdh *(Glyceraldehyde 3-phosphate dehydrogenase) mRNA levels and represent averages and SD from direct comparison of LY294002 and DMSO treatments from at least 3 different trials.

### Organ culture

Tibiae were isolated from E15.5 mice and cultured for 6 days in medium containing alpha MEM, ascorbic acid, beta-glycerophosphate, bovine serum albumine, glutamine and Penicillin-Streptomycin, as described [13]. After dissection, the bones were incubated in this medium over night and then treated with LY294002 (10 μM), PI3-K α inhibitor IV (500 nM) or DMSO. In the case of IGF1 treatments the tibiae were cultured in the presence of control (0.1% Bovine serum albumine (BSA) in PBS with DMSO), IGF1- 50 ng/ml- (with DMSO), LY294002 (with 0.1% BSA) or IGF1+LY294002. The control for CNP was 0.1% HCl-BSA in PBS and the CNP concentration used, 10^-6 ^M. The treatments were organized similar to IGF1. Media and supplements were changed every two days. The bone length was measured at the beginning (before any treatment) and end of the time course. After 6 days of treatment, organs were fixed and paraffin embedded. 5 μm sections were stained with Hematoxylin and Eosin (H&E), Safranin O/Fast green and Alcian blue and then analyzed using a Leica DMRA2 microscope. Whole tibiae were also stained with Alcian blue/Alizarin red S.

### Histology and immunohistochemistry

Histology and immunohistochemistry procedures were performed as described [50] with minor modifications. Sections were incubated in 3% H_2_O_2 _for 15 min at room temperature, followed by boiling for 2 min and incubation for 30 min at 97°C in 10 mM sodium citrate (pH 6.0) or pronase E treatment (1 mg/ml of PBS) for 10 min in the case of collagen X. They were then blocked with 5% goat serum. Sections were incubated with primary antibodies over night at 4°C. The UltraVision LP Large Volume Detection System AP Polymer was then used to recognize the primary antibodies according to manufacturer's instructions. After washing, the HRP (horseradish peroxidase) conjugated polymer complex was visualized by incubation for 2–10 min with AEC (3-amino-9-ethylcarbazole) substrate-chromogen and then sections were washed and mounted. All images were taken at room temperature with a Retiga EX camera connected to a Leica DMRA2 microscope. Primary image analyses were performed using Openlab 4.0.4 software.

### TUNEL assay

The bones were cultured for 6 days in the presence of LY294002 or DMSO, fixed in 4% Paraformaldehyde and paraffin embedded. 5 μm sections were used for the TUNEL assay using the Calbiochem^® ^DNA Fragmentation Detection Kit according to manufacturer's instructions.

### Statistical analysis

All data were collected from at least 3 independent trials, which were run in triplicate or quadruplicate. Data were expressed as mean ± SD, and * p values under 0.05 were considered significant (*). Statistical significance was determined by one-way ANOVA (for Figure 2B) and two-way ANOVA (for the rest of the graphs), with Bonferroni post-test using GraphPad Prism 3.00 for Windows.

## Authors' contributions

VU performed most experiments and contributed to study design and the writing of the manuscript. KH and JRG performed selected experiments. FB contributed to study design and the writing of the manuscript. All authors read and approved the submitted version of the manuscript.
